# Six types of loves differentially recruit reward and social cognition brain areas

**DOI:** 10.1093/cercor/bhae331

**Published:** 2024-08-26

**Authors:** Pärttyli Rinne, Juha M Lahnakoski, Heini Saarimäki, Mikke Tavast, Mikko Sams, Linda Henriksson

**Affiliations:** Department of Neuroscience and Biomedical Engineering, Aalto University, Rakentajanaukio 2 C, 02150 Espoo, Finland; AMI Centre, Aalto NeuroImaging, Aalto University, Magnet house, Otakaari 5 I, 02150 Espoo, Finland; Institute of Neuroscience and Medicine, Brain & Behaviour (INM-7), Research Center Jülich, Wilhelm-Johnen-Straße, 52428, Jülich, Germany; Institute of Systems Neuroscience, Medical Faculty, Heinrich Heine University Düsseldorf, Moorenstr. 5, 40225, Düsseldorf, Germany; Faculty of Social Sciences, Tampere University, City Centre Campus Linna building, 6. floor., Kalevantie 5, 33014 Tampere, Finland; Department of Neuroscience and Biomedical Engineering, Aalto University, Rakentajanaukio 2 C, 02150 Espoo, Finland; Department of Computer Science, Aalto University, Computer science building, Konemiehentie 2, 02150 Espoo, Finland; Department of Neuroscience and Biomedical Engineering, Aalto University, Rakentajanaukio 2 C, 02150 Espoo, Finland; MAGICS–Aalto, Aalto University, P.O. Box 11000 (Otakaari 1 B), Finland; Department of Neuroscience and Biomedical Engineering, Aalto University, Rakentajanaukio 2 C, 02150 Espoo, Finland

**Keywords:** close relationships, emotion, fMRI, limbic system

## Abstract

Feelings of love are among the most significant human phenomena. Love informs the formation and maintenance of pair bonds, parent-offspring attachments, and influences relationships with others and even nature. However, little is known about the neural mechanisms of love beyond romantic and maternal types. Here, we characterize the brain areas involved in love for six different objects: romantic partner, one’s children, friends, strangers, pets, and nature. We used functional magnetic resonance imaging (fMRI) to measure brain activity, while we induced feelings of love using short stories. Our results show that neural activity during a feeling of love depends on its object. Interpersonal love recruited social cognition brain areas in the temporoparietal junction and midline structures significantly more than love for pets or nature. In pet owners, love for pets activated these same regions significantly more than in participants without pets. Love in closer affiliative bonds was associated with significantly stronger and more widespread activation in the brain’s reward system than love for strangers, pets, or nature. We suggest that the experience of love is shaped by both biological and cultural factors, originating from fundamental neurobiological mechanisms of attachment.

## Introduction

Feelings of love are among the most salient in human life: they may provide intense pleasure while promoting pair bonding and parental investment (Bartels and Zeki 2000; Bartels and Zeki 2004; Nummenmaa et al. 2014; Nummenmaa et al. 2018; Rinne et al. 2023). Previous studies suggest that feelings of romantic and maternal love are associated with activation of attachment and reward networks in the brain (Bartels and Zeki 2000; Bartels and Zeki 2004; Aron et al. 2005; Fisher et al. 2005; Noriuchi et al. 2008; Acevedo et al. 2012; Shih et al. 2022). These evolutionarily old brain regions have been shown to be involved in both long-term bonding and parental care behaviors also in other mammals (Winslow et al. 1993; Bartels and Zeki 2004; McGraw and Young 2010; Tabbaa et al. 2016). But when we love, is it neurally the same thing to love, for instance, our child as to love nature?

Even though romantic and parental love form the prototypical and biological core of love, the human phenomenon of love is much more. Psychological, philosophical, and theological conceptualizations of love abound with various taxonomies, often offering rich vocabularies that permit love to be felt for people beyond one’s immediate family—think of love for one’s friends and love for strangers (or “neighbors,” as strangers are often called in Christian parlance). Complex, historically resilient social and cultural institutions concerning billions of people are built on notions involving transcendent entities that allegedly feel love for the whole of humankind—or at least for a particular ethnic or sociocultural subgroup. Human love may transcend boundaries between species, as pet owners feel and express love for their pets, and mutual gazing between dogs and their owners has been found to engage oxytocin pathways similarly to mother–infant bonding (Nagasawa et al. 2015, see also Applebaum et al. 2021). Feelings of love may not even require individual organisms or beings as their counterparts, as a recent study found that love of nature is among the most often experienced types of love (Rinne et al. 2023). Objects of love are socially, culturally, and subjectively variable (see Fehr and Russell 1991; Fehr 1994; Shpall 2016; cf. Rinne et al. 2023). Subjective feelings of love for various objects form a continuum from strongly to weakly felt loves (Rinne et al. 2023).

Love is closely linked to feelings and behaviors related to attachment. Even though the concept of attachment is often associated mainly with pair bonding and/or parental care, the human phenomenon of attachment covers a wider array of relations and objects. In her recent theorization of the neurobiology of human attachments, Feldman treats the neurobiology of attachment bonds as synonymous with that of love (Feldman 2017). With respect to mammals, she classifies these bonds into parent–infant, pair bond, peer (friend), and conspecific (unknown member of the same species) relations according to degrees of social proximity and biobehavioral intimacy. In this conceptualization, the term “attachment” cannot be reduced to pair bonding or parent-offspring relations but is a generic term informing various gradients of affiliation such that affiliations with conspecifics (strangers) represent the weakest degree of affiliation. In their state-of-the-art meta-analysis of neuroscientific research on human affiliation, Bortolini et al. (2024, 2) adopt the view that the terms “affiliation,” “bonding,” and “attachment” may be treated synonymously because of conceptual overlap. These authors define “affiliation” as “one’s disposition to enjoy, seek, and sustain close interpersonal bonds. On a subjective level, it involves feelings of warmth and affection for significant others.” Here, we adopt the gradient typology of Feldman (2017), according to which affiliation comes in degrees according to social closeness.

The conceptual classifications concerning human affiliative neuroarchitecture remain under debate. Feldman (2017) proposes a “global human attachment system” consisting of three partially overlapping brain networks: (i) *reward-motivation system* (striatum [nucleus accumbens, caudate, putamen], amygdala, ventral tegmental area, orbitofrontal cortex, ventromedial prefrontal cortex, and anterior cingulate cortex); (ii) *embodied simulation/empathy network* (insula, anterior cingulate cortex, inferior frontal gyrus, inferior parietal lobule, and supplementary motor area); (iii) *mentalizing system* (superior temporal sulcus, posterior cingulate cortex, temporoparietal junction, temporal pole, and medial prefrontal cortex). Based on the meta-analysis of 79 fMRI studies, Bortolini et al. (2024) classify their results of closer affiliative bonds (parent–infant, romantic, friend) into areas related to (i) *social reward* (ventral pallidum, thalamus, striatum, ventromedial prefrontal cortex, medial preoptic area, and septo-hypothalamic region); (ii) *social cognition* (extended amygdala, posterior cingulate and precuneus, anterior insula, inferior parietal lobule, and inferior frontal gyrus); (iii) *salience* (amygdala, anterior cingulate cortex, and insula). Based on previous research, we take it that the distinctions between networks associated with reward, motivation, and salience on the one hand, and social cognition on the other, are sufficiently well established and robust to serve as conceptual axes of analysis in a neuroscientific study, where the stimuli aim to manipulate degrees of interpersonal social closeness and social/nonsocial context. We use the term “reward brain areas” to refer to areas often associated with reward, motivation, and salience, and the term “social cognition brain areas” to refer to the areas associated with social cognition including embodied simulation, empathy, and mentalizing or “theory of mind.”

Despite the growing number of studies of human affiliation, till today, very little is known about the neural mechanisms associated specifically with feelings of love beyond the romantic and maternal types (cf. Beauregard et al. 2009). The seminal experiments of Bartels & Zeki (Bartels and Zeki 2000; Bartels and Zeki 2004) in the early 2000s were followed by a brief period of “normal science” (see Kuhn 1996), where subjects were shown photographs of the faces or videos of their romantic partners or their infant-age children, while the hemodynamic activity of their brain was measured with fMRI. Overall, the neuroscience of love remains considerably understudied. Given the significance of the topic, and newer theorizations of love (Fredrickson 2016; Feldman 2017), which extend beyond romance and maternity, we argue that the study of neural mechanisms of different types of love is ripe for conceptual and methodological expansion.

Here, we investigated the neural activity during feelings of love for six different types of objects: romantic partners, one’s own children, friends, strangers (varieties of interpersonal love), nonhuman pets (interspecies love), and nature (nonsocial love). We chose the categories of the types of love to include the most prototypical and most researched types of interpersonal love related to pair bonds and parental care (romantic and parental love). We also included subjectively less salient yet culturally and linguistically prototypical types of interpersonal love for peers and nonfamiliar conspecifics (love for one’s friends, compassionate love for unknown people commonly called “neighborly love” in the Christian cultural sphere). Together these four types of interpersonal love form a scale of interpersonal affiliative bonds from stronger to weaker affiliations following Feldman’s typology (see Fehr and Russell 1991; Feldman 2017; Rinne et al. 2023). Extending conceptually beyond interpersonal relations, we further included the category of nonhuman pets to probe the neural correlates of interspecies love, and a nonsocial category of love for beautiful nature to compare the neural correlates of a frequently experienced type of nonsocial love (see Rinne et al. 2023) with those of social love.

Feelings of love were induced using short, spoken, pre-recorded stories during fMRI scanning. Narratives have been shown to be a powerful means of emotion induction (Jääskeläinen et al. 2021; Saarimäki 2021). Each narrative depicted an everyday situation eliciting the feeling of love for one type of object. We also included neutral control stories about mundane situations where nothing special happens. To allow for a distinction between the neural activation in the simulated phenomenological situation (audio story) and immersion in the feeling elicited by the situation, we included an imagery period, which followed each story (see Saarimäki et al. 2018). During the imagery period, the task of the participants was to immerse themselves in the feeling elicited by the narrative.

We asked: which brain areas are activated during feelings of love? Which areas are common to feelings of love for different types of objects and how do the areas differ based on the type of object? Based on previous research (see Ortigue et al. 2010; Shih et al. 2022; Rinne et al. 2023), we hypothesized that the activation of the subcortical reward system is a common substrate underlying various feelings of love. We also hypothesized that the feelings of love for different objects might be differentially modulated by social brain networks, and that the degree of reward system activation could be associated with the closeness of the affiliative bond in question. Furthermore, our study may shed additional light on the big question of why humans apply the word “love” to a wide variety of situations.

## Materials and methods

### Subjects

Fifty-five subjects (29 females, 26 males) participated in the fMRI experiment. We recruited native Finnish speaking, healthy adults with no regular medication, participant age range 28 to 53 (mean: 40.3), who self-reported to have at least one child and to currently be in “a loving couple-relationship” (mean duration of relationship: 11.9 years). The subjects were recruited through email lists and public social media groups in the metropolitan area of Helsinki, Finland. 27 subjects were pet owners. The study was performed to ethical standards as laid down in the 1964 Declaration of Helsinki and its most recent 2013 revision and was approved by the Aalto University Research Ethics Committee (D/461/03.04/2021). The subjects gave informed consent prior to the experiment.

### Stimuli

The different types of love were induced by short prewritten and prerecorded audio narratives (three sentences; duration ca. 13–15 s/narrative) (cf. Saarimäki et al. 2018). The narratives depicted everyday situations. Each narrative described a scenario with one object of love. The categories of objects of love were one’s romantic partner, one’s child, one’s friend, a pet (dog or cat), a stranger, and the surrounding (beautiful) nature. We also included a neutral control category. There were six narratives per category totaling 42 narratives (36 love narratives and six control narratives).

One day prior to scanning, the subjects were informed in a telephone call that they would be listening to short narratives about love during the fMRI experiment. To facilitate emotion induction in fMRI, the subjects were asked to take 5 to 10 mins on the eve of the experiment to think about and dwell affectively in what love feels like for them personally with respect to the six types of objects of love in the study. The different objects of love were merely named in the phone call and no further qualifications of semantic or emotive content of love was imposed on the subjects. At the laboratory, the subjects received instructions to “immerse themselves [Finnish: eläytyä] in the depicted scenario as vividly as possible,” and to “do their best to imagine what love would feel like if they were in the given situation.” The love narratives always ended with the prompt “You feel love for [x] / You love [x],” for example: *You are in the laundry room with your partner. They are loading the washer with laundry, and suddenly you remember what a lovely person your partner is. You feel love for them.* Or: *Your child runs to you joyful on a sunny meadow. You smile together and the sunrays flicker on their face. You feel love for your child.*

Love has been previously identified as a positive feeling with similarities to, for example, happiness, pleasure, and togetherness (see Nummenmaa et al. 2018). Feelings of interpersonal love are mediated through biobehavioral synchrony including smiling (Fredrickson 1994; Fredrickson 2016). These generic qualities of loving feeling were reflected in the narratives. Even though the behaviors associated with love between living beings often involve touching, we wished to avoid the potential confound related to touch and thus omitted the dimension of touch from the stories. The stories about love for one’s friends included depictions of reciprocal everyday altruism and sharing thoughts and feelings, for example: *You need help moving house and you call your friend. They promise to of course come to help out, and soon you are lifting cardboard boxes together in a van. In the middle of the ordinary situation you feel love for your friend.* The stories depicting compassionate love for unknown people involved acts of everyday benevolence from the subject to the object of love, reciprocated with an expression of gratitude: *You see an old woman on the street carrying heavy grocery bags. You help her by carrying one of the bags to her home door one block away. The old woman is grateful, and you feel love for her.* The stories concerning love for pets were divided into three stories about dogs and three stories about cats; e.g. *You are in a park playing with your dog. You toss a stick for the dog and it retrieves it enthusiastically wagging its tail. You love your dog.* The nonsocial stories concerning love for nature depicted beautiful natural surroundings, in which the subject found themselves immersed: *You are in the archipelago at the seaside. The blue waves ripple over the coastal stones, a crooked pine rises next to you, and there are white fluffy clouds here and there in the sky. You love nature.* The neutral stories involved a mundane everyday situation where nothing special happens, and there is no social interaction: *You are on the bus going home. Through the window of the bus one can see houses, cars, and people walking on the street. The view is quite ordinary* (for all narrative stimuli with accompanying English translations in parallel text format, see supplementary information Stimuli).

The narratives were written by a professional scriptwriter (P.R.) and recited by a professional actor (female in her early forties), who was instructed to read the narratives out loud in a neutral, natural tone without emphasizing any emotion but not sounding mechanical either.

### fMRI data collection procedure

The stories were grouped into six runs of seven stories, such that each run included one story from each category. The order of the stories was randomized for each subject. The subjects listened to the stories through MRI-compatible earphones (Sensimetrics S14). The timing of the stimulus presentation was controlled with Presentation software (Neurobehavioral Systems, Albany, CA).

In the MRI scanner, a soundcheck was performed, such that the subjects confirmed that they can hear the narratives and the volume is comfortable for them with the scanner on. Before the audio runs, the subjects were shown a 6-min animation film Partly Cloudy while being scanned (data not reported here). The audio runs began with a written instruction on the screen for the subject to close their eyes. Each trial within a run consisted of the audible narrative, followed by 10 s of silence during which the task of the subject was to immerse themselves in the feeling elicited by the narrative. The 10-s immersion task ended with a beep followed by a 10-s washout period, for which the subjects received instructions prior to the experiment to “let their minds revert and become empty of the previous emotion.” Together with the randomization of presentation order, this washout period controlled for any systematic effects left over from the previous condition. The experimental design followed the paradigm used in a previous study (Saarimäki et al. 2018). Individual runs ended with three beeps, which cued the subjects to open their eyes. Between the runs, the subjects responded to two questions shown on the screen by pressing buttons with their fingers on a four-point scale: “How easy was it for you to immerse yourself in the stories?”; “How loving do you currently feel in relation to the world in general?” Our rationale for imposing these questions was to control that the subjects are awake and alert to the experiment between runs.

The functional and structural MRI data were acquired using a 3 T MAGNETOM Skyra whole-body scanner (Siemens Healthcare, Erlangen, Germany) equipped with a 30-channel head coil. The whole-brain functional volumes were acquired using a T2*-weighted simultaneous multislice (SMS; multiband factor 4) echo-planar imaging (EPI) sequence with imaging parameters: repetition time 852 ms, 60 slices with 3 mm slice thickness (no gap), field of view 192 × 192 mm^2^, imaging matrix 64 × 64, echo time 30 ms, and flip angle 55°. T1-weighted structural images with a spatial resolution of 1 × 1 × 1 mm^3^ were collected using a T1-weighted MPRAGE sequence.

### Behavioral ratings and other supplementary data

Besides fMRI, we collected various supplementary data. Prior to scanning, the subjects responded to the 15 item Passionate Love Scale questionnaire (Version A) (Hatfield and Sprecher 2011) and the Shortened 9 item Compassionate Love Scale for Humanity questionnaire (Chiesi et al. 2020, see Sprecher and Fehr 2005). Before and after scanning, the subjects further responded to the question “How loving do you currently feel in relation to the world in general?” on a 1 to 9 scale. We also collected the subjects’ salivary samples from three time points: immediately before entering the scanner, immediately after scanning, and 20 mins from the second measure. We aimed to analyze the oxytocin concentration from these samples, but based on preliminary analysis, the data quality was deemed low due to problems either in saliva collection or laboratory analysis of the samples.

After the scanning, the subjects performed two tasks where they provided ratings for the seven stimulus categories. During both tasks, the participants were given a sheet of paper consisting of textual versions of the stimuli. The six stimuli in each category were organized under one headline (the category name), and the participants were allowed to read and recall the stories during the tasks, without time limit.

In the first task, the participants were asked to evaluate the overall feelings elicited by each of the seven stimulus categories during the fMRI scanning. They rated their experiences in terms of seven dimensions: (i) “How strongly does the feeling elicited by the stories feel in the body” (*not at all—very much*). (ii) “How strongly does the feeling elicited by the stories feel in the mind” (*not at all—very much*). (iii) “How pleasant was the feeling elicited by the stories” (*extremely unpleasant—extremely pleasant*). (iv) “How arousing was the feeling elicited by the stories” (*calm—excited*). (v) “How often do you feel a feeling that is similar to the one elicited by the stories?” (*never—often*). (vi) “How well do the feelings elicited by the stories correspond with your own conception of what love is?” (*not at all—extremely well*). (vii) “How easy was it for you to immerse yourself in the situations described in the stories?” (*extremely hard—extremely easy*). The participants were shown each stimulus category one-by-one on a computer screen, and their task was to evaluate the experience by marking their answer on a horizontal line (i.e. visual analogue scales, minimum value 0, maximum value 1,000). The stimuli were presented in random order.

In the second task, the participants were asked to rate how similar are feelings associated with the six love types and neutral feeling on a 7-point scale from “extremely different” to “extremely similar.” They rated each of the possible 21 pairwise comparisons one-by-one. For each participant, the order of the pairwise comparisons was randomized. The task was conducted using paper and pen.

We also conducted a separate online study with different participants (*n* = 181), where ratings for the seven scales of task 1 were collected separately for textual versions of all the 42 stimuli used in the fMRI experiment. The results of the online study are presented in the supplementary materials (see Supplementary Fig. S3; Supplementary Table S2).

### Functional MRI data analysis

Functional MRI (fMRI) data were preprocessed with SPM12 MATLAB toolbox (http://www.fil.ion.ucl.ac.uk/spm, Wellcome Trust Centre for Neuroimaging, London, United Kingdom). The functional images were corrected for head motion, normalized to Montreal Neurological Institute (MNI) standard space, and spatially smoothed using a 6-mm Gaussian smoothing kernel. The responses for each love category and the neutral control category were estimated using standard general linear model (GLM) analysis as implemented in SPM12. Regressors of interest for each love category and the neutral condition were constructed based on the onsets and durations of the narratives. In addition, regressors for the mental imagery of each condition were time-locked to the end of the narratives. The six head motion parameters and the timings of the bleeps, which were used as cues for the subject during the scanning, were included as nuisance regressors. Each regressor of interest was convolved with the canonical hemodynamic response function. During parameter estimation, the data were high-pass filtered with a 128-s cut-off. In the first-level analysis, individual main effect maps for audio and imagery condition were produced contrasting the level of activity during each stage of the experiment to the baseline (mean of unmodeled time points). Additionally, contrast maps were created for each love category contrasting them with the neutral control condition. The resulting first-level maps were subjected to second-level analyses in the statistical nonparametric mapping (SnPM; http://warwick.ac.uk/snpm) extension in SPM12. Furthermore, we calculated parametric contrasts between all pairs of the six different types of love during audio and imagery conditions, leading to 30 pairwise contrasts. Voxel-level threshold was set to *P* < 0.001, uncorrected, with cluster-level correction of the family-wise error rate (FWER) at *P* < 0.05 in all whole brain analyses.

To summarize and annotate the locations of significant activation clusters, we used the Brainnetome atlas (Fan et al. 2016) for the cerebral cortex, probabilistic atlas of the cerebellum (Diedrichsen et al. 2009; Diedrichsen et al. 2011) for cerebellar regions and Brainstem navigator (Bianciardi 2021; see Bianciardi et al. 2015; Bianciardi et al. 2018; García-Gomar et al. 2019; García-Gomar et al. 2021; Singh et al. 2020; Singh et al. 2021) for the brainstem and diencephalon. Custom code employing the spm_clusters function was used to extract the contiguous activation clusters and summarize the number of voxels in each atlas region that were included in the significantly activated cluster. In the summary matrix visualization, we included only those atlas regions of which at least 5% was significantly activated, by volume (voxel count). For the brainstem matrices in the supplementary results, we included only those nuclei with > 10% volume activated due to the small size of the regions. The Supplementary Tables file includes the complete lists of local activation maxima and the percentage of volume of atlas regions included in each significant activation cluster for the contrasts of each love category against neutral stories in a separate sheet. First seven sheets report the main effects for categories of love and the control condition during the audio story, followed by the six love categories contrasted with the control condition. The last thirteen sheets report analogous results for the imagery condition. Each sheet lists first the positive clusters followed by the negative clusters and reports the cluster sizes, local extreme values, MNI coordinates, and the names and percentage of significant voxels and MNI coordinates of atlas regions that the cluster (partially) overlaps with.

## Results

### Questionnaire and behavioral tasks results

The mean score for the Passionate Love Scale was 6.83 (standard deviation 0.69, range 5.60–8.47). On average, the participants were passionately in love with their partners (see Hatfield and Sprecher 2011). For the Compassionate Love Scale for Humanity, the mean score was 4.87 (standard deviation 0.97, range 2.11–6.78). The participants further evaluated how loving they feel in relation to the world in general before and after fMRI (scale 1–9). The mean score for love for the world in general was 6.8 (standard deviation 1.069, range 4–9) before fMRI and 7.75 (standard deviation 0.86, range 5–9) after fMRI.

**Fig. 1:**
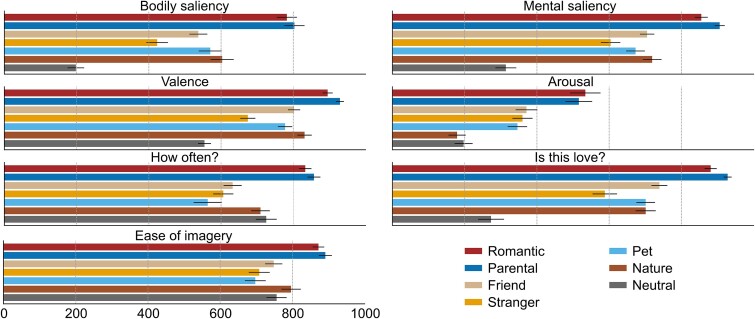
Means (± standard error of the mean) of the seven rating dimensions for the stimulus categories on a scale from 0 to 1,000.

After the fMRI measurement, the participants also rated their overall feelings elicited by the stimulus categories (Fig. 1). *Romantic* and *parental love* were the two highest rated categories in all seven dimensions. Of the other love types, *love for strangers* was rated to correspond least with the participants’ own understanding of what love is. *Love for pets, love for strangers,* and *love for friends* were the three most difficult categories to be immersed in during the imagery task.

**Fig. 2:**
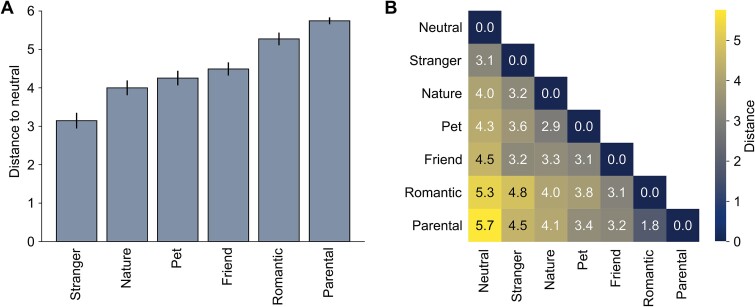
Similarity of feelings associated with stimulus categories. A) Mean distances to neutral feeling for all the love types from the similarity of feelings rating task. Smaller distance (lower values) represents higher similarity. The error bars show standard errors of the means. B) Mean distances between all the stimulus categories. Distances range from 0 to 6.

In addition, the participants evaluated the similarity of the feelings associated with the stimulus categories through pair-wise comparisons. The results are presented in Fig. 2, with similarities converted to distances. The most distant love type from *neutral feeling* was *parental love,* followed by *romantic love* and *love for friends*. Of the love categories, *love for strangers* was the least different from the neutral feeling. *Love of nature* was rated to be as distant from *neutral feeling* as from *romantic love,* and even slightly more distant from *parental love* than from *neutral feeling* (see Fig. 2B).

### Brain regions associated with “love in general”

**Fig. 3:**
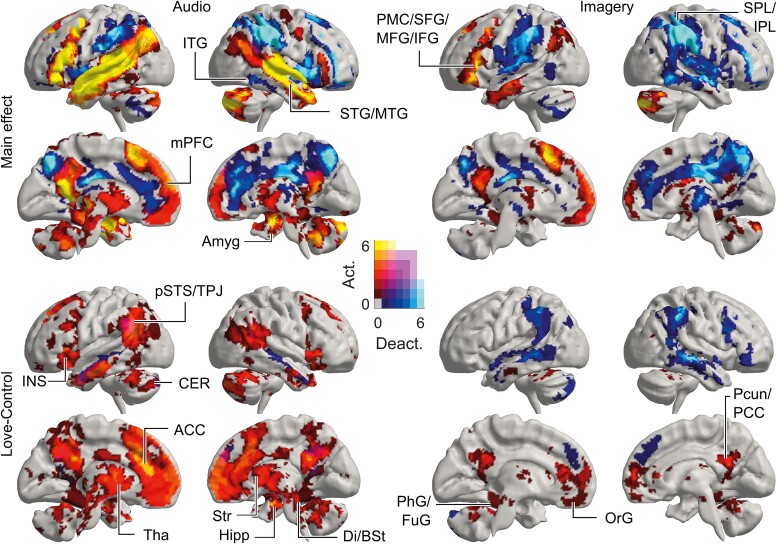
Number of overlapping categories of love as activating and deactivating brain regions. “Main effect” depicts the overlap of love categories as contrasted against BOLD-signal baseline, whereas “love-control” shows the overlap when contrasted against the neutral control condition. “Audio” refers to activations during the listening of the narratives, whereas “imagery” shows the activations during the 10 s of silence, which followed the prompt “you feel love for [x]” at the end of each narrative. Hot colors correspond to activations and cold colors to deactivations. Areas where a subset of categories activated, and others deactivated the same region are shown in mixed colors (purple shades). If the image is not accessible due to colors, please see Supplementary Tables. For abbreviations, see main text and Fig. 4.

Figure 3 shows the overlap of activations for all types of love. Upper panels of Fig. 3 depict how many love categories activated or deactivated each brain area during listening to the stories (left) and during the imagery period that followed each story (right) compared to baseline between trials. All love stories activated large areas in the bilateral temporal lobes and left prefrontal cortex as well as cerebellum (likely reflecting the lexical processing of the narrative irrespective of its emotion content; see also Supplementary Fig. S1 for activation elicited by the neutral story). Activations during audio stories were more widespread than during imagery whereas more deactivations were identified during imagery than during stories. Most of the love stories deactivated large areas in the parietal and temporal cortices, as well as the medial cortical areas. When investigating the temporal evolvement of these deactivations, we found that they began almost immediately after auditory responses and lasted longer into the imagery period than the activations.

**Fig. 4:**
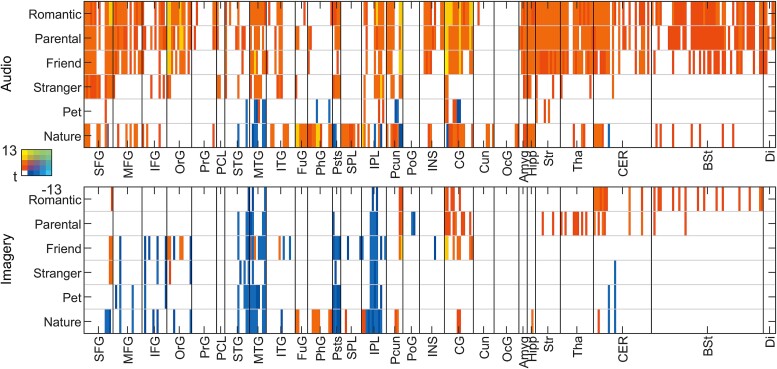
Summary matrix of activity at the level of brain regions against control. Columns show the extreme *t*-values of significant voxels for all regions where at least 5% of voxels within the region were significantly (de)activated (*P*_voxel_ < 0.001, *P*_cluster_ < 0.05, FWER). Positive effects are shown in hot and negative effects in cold colors. Areas containing both positive and negative effects are indicated by mixed colors (purple to green). If the image is not accessible due to colors, please see Supplementary Tables. Abbreviations: SFG: superior frontal gyrus; MFG: middle frontal gyrus; IFG: inferior frontal gyrus; OrG: orbital gyrus; PrG: precentral gyrus; PCL: paracentral lobule; STG: superior temporal gyrus; MTG: middle temporal gyrus; ITG: inferior temporal gyrus; FuG: fusiform gyrus; PhG: parahippocampal gyrus; Psts: posterior superior temporal sulcus; SPL: superior parietal lobule; IPL: inferior parietal lobule; Pcun: precuneus; PoG: postcentral gyrus; INS: insular gyrus; CG: cingulate gyrus; Cun: cuneus; OcG: occipital gyrus; Amyg: amygdala; Hipp: hippocampus; str: striatum; Tha: thalamus; CER: cerebellum; BSt: brainstem; Di: diencephalon.

Because part of these activations and deactivations are likely to be explained by the auditory processing of the stories, we next contrasted all love conditions against the control condition (neutral story) and will focus on these contrasts in the following parts of the manuscript. As shown in the bottom left panel of Fig. 3, we found activations especially in the medial prefrontal cortex (mPFC), orbitofrontal cortex/gyrus (OrG), anterior and posterior cingulate cortex (ACC, PCC), right middle temporal gyrus (MTG), posterior superior temporal sulcus/temporoparietal junction (pSTS/TPJ), precuneus (Pcun), insula, subcortical regions (amygdala, hippocampus, striatum, thalamus), and cerebellum. Across all types of love, we found activations in superior frontal gyrus (SFG), inferior parietal lobule (IPL), and cingulate gyrus (CG) (see Fig. 4). Note that ACC was deactivated by all stories, and the deactivation was less for love compared to neutral control condition (see Supplementary Fig. S1 for all main effects). We also found activation in brainstem regions, including areas in midbrain, diencephalon, pons, and medulla (Supplementary Fig. S2). During the imagery period, we found activations in medial frontal cortex, orbitofrontal cortex, anterior and posterior cingulate cortices, and precuneus when contrasted to the neutral control condition (Fig. 3 “Love-Control,” bottom right panel). Compared to the main effects of audio and imagery periods, activations for the love conditions vs. neutral control condition were smaller in inferior frontal gyrus and temporal cortex, which suggests that these regions were mostly explained by auditory processing, and more widespread in medial prefrontal cortex, insula, and subcortical regions, which supports the role of these regions in the processing of love-related content.

### Brain regions associated with different types of love

**Fig. 5:**
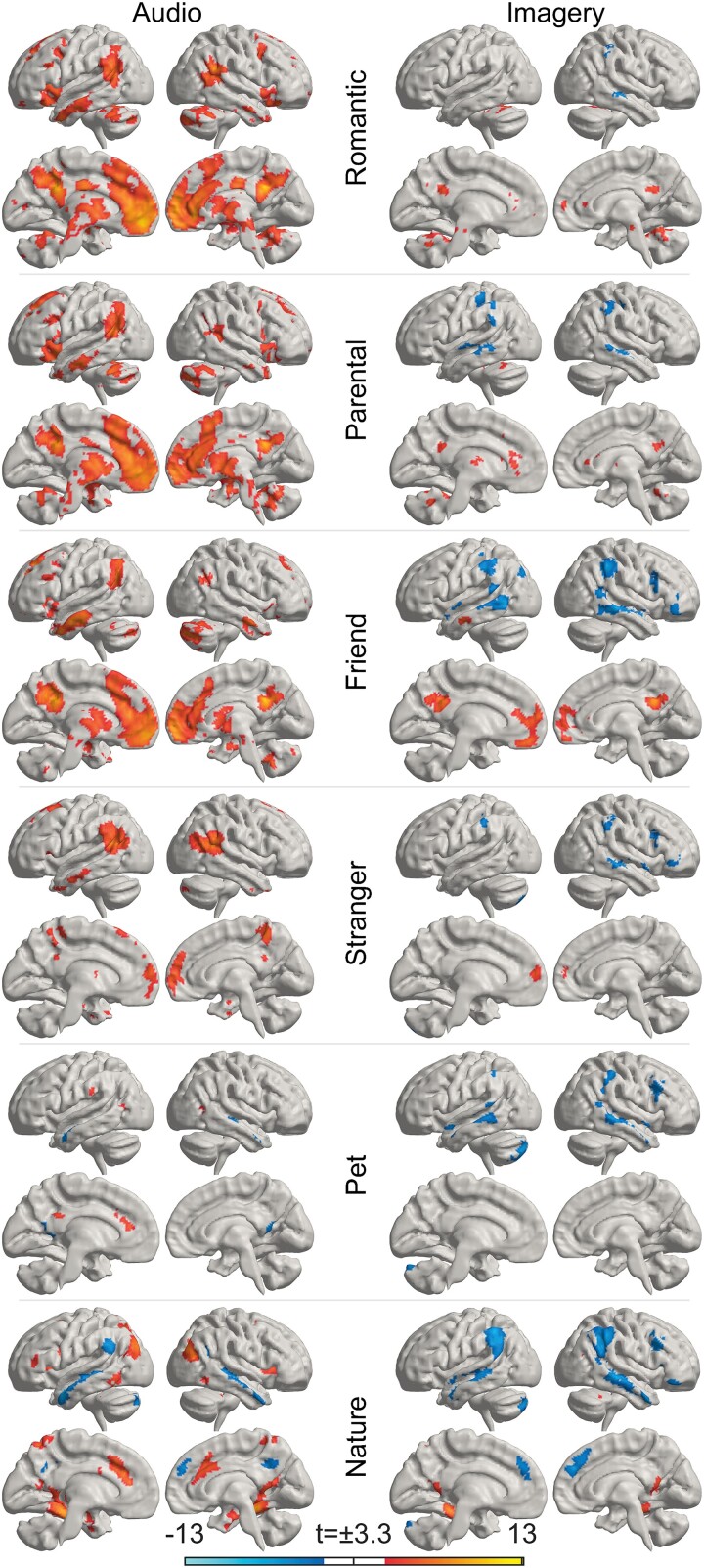
The brain regions activated and deactivated while listening to stories of love (left) and imagining (right) feelings of love for different objects as contrasted with neutral control stories (*P*_voxel_ < 0.001, *P*_cluster_ < 0.05, FWER). Hot colors indicate areas that were activated more strongly for love than control stories. Cold colors indicate areas that were activated more strongly for control than love stories or were deactivated more strongly for love than control stories (for main effects contrasted against bold-signal baseline, see Supplementary Fig. S1). If the image is not accessible due to colors, please see Supplementary Tables.

Next, we examined the activations related to different types of love separately as contrasted to the neutral control condition (Figs 4 and 5; for main effects of each category contrasted against BOLD-signal baseline, see Supplementary Fig. S1. Cluster statistics are reported in the Supplementary Tables file). Overall, feelings of love for different objects were associated with similar, yet partially distinct patterns of activation. The activation of different brain regions with respect to different types of love appeared to be modulated especially by the closeness of the affiliative bond in question and whether the feeling of love involved an interpersonal or noninterpersonal context. Notably, during audio stories, the most widespread love-related activation was observed for closest relationships (romantic love, parental love, and love for friends) in insula, striatum, thalamus, and brainstem. Activations were also found in cerebellum, midline regions (including ACC, PCC, and Pcun), and medial frontal cortices (including SFG, MFG, OrG). During the imagery period, we again found most activation in relation to closest relationships in cingulate cortices, precuneus, subcortical regions including striatum, thalamus, brainstem, and cerebellum. However, these activations were less widespread than activations during the audio stories.

For *romantic love* as contrasted to the control condition (Fig. 5, uppermost left panel), we found activation during audio stories especially in medial superior and middle frontal gyri, orbitofrontal gyrus, temporal regions (including superior, middle, and inferior temporal gyri), posterior superior temporal sulcus (pSTS), anterior and posterior cingulate cortices, insula, subcortical regions (amygdala, hippocampus, striatum, thalamus), and in cerebellum. In addition, we found widespread activation also in the brainstem covering nuclei in midbrain, diencephalon, pons, and medulla (Supplementary Fig. S2). During imagery, activations were found in parts of medial frontal lobe, posterior cingulate, precuneus, and cerebellum. In brainstem, activations were found mainly in the midbrain.

For *parental love*, we found activations in largely the same brain regions as for romantic love especially during audio stories. Notably, during imagery of parental love, we found activation in striatum and thalamus; this activation was not found for other types of love.

For *love for friends*, we again found activation during audio stories especially in medial superior and middle frontal gyri, orbitofrontal gyrus, pSTS, temporal regions (including superior, temporal, and inferior gyri; though less widespread than for closer relationships), cingulate cortices and precuneus, insula, subcortical regions including amygdala, striatum, thalamus, and cerebellum. In brainstem, activations were located mostly in the midbrain and diencephalon. During imagery, activations were found in medial superior and orbitofrontal gyri, precuneus, and posterior cingulate cortex.

For *love for strangers*, we found less activation than for the closer relationships. During audio stories, activation was identified in medial superior and orbitofrontal gyri, pSTS, temporal regions, and amygdala. We also found activation in cingulate cortex, parts of striatum, and thalamus, but these activations were smaller than for closer relationships. During imagery, activations were observed only in parts of medial superior and orbitofrontal gyri.

**Fig. 6:**
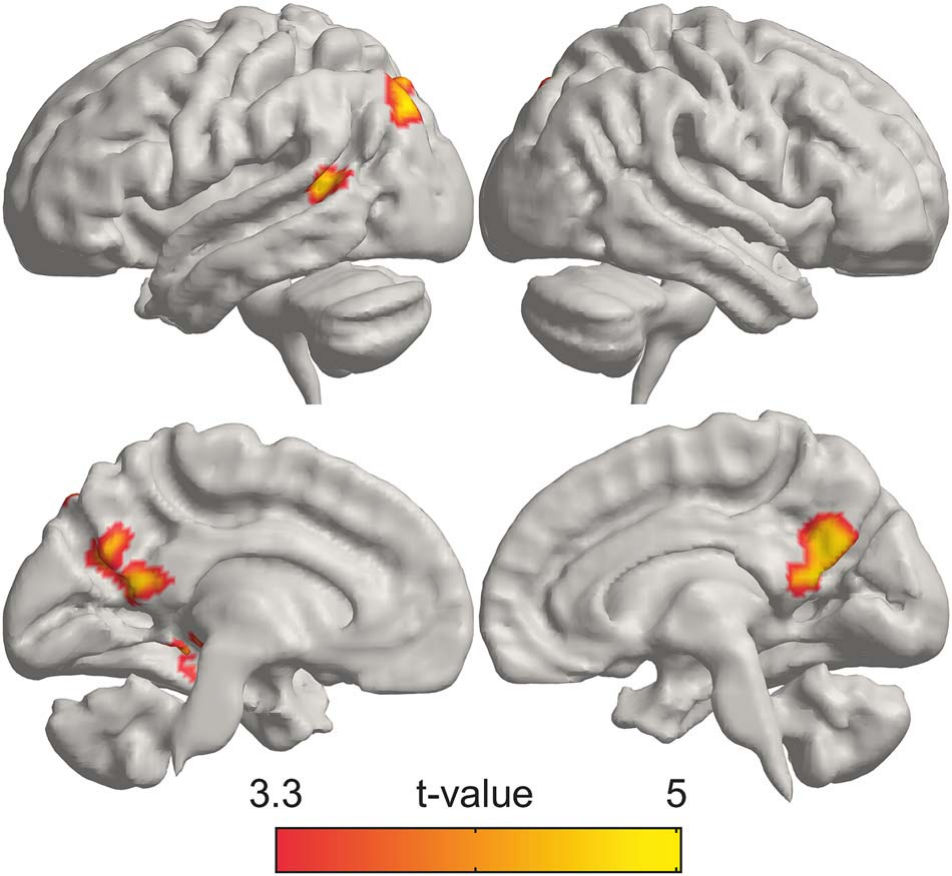
Brain regions activated more strongly in pet owners than participants without pets in love for pets vs. control contrast (*P*_voxel_ < 0.001, *P*_cluster_ < 0.05, FWER).

For *love for pets*, we found overall less activation than for love for humans. We identified activation during audio stories in small parts of superior frontal gyri, pSTS, posterior cingulate cortex, and striatum. During imagery, we observed activations in small parts of medial frontal cortex, including superior and orbitofrontal gyri and ACC. In participants who had pets compared with participants without pets, the activity was significantly higher in the precuneus/posterior cingulate cortex, pSTS, inferior parietal lobule, and ventral temporal lobe including parts of fusiform and parahippocampal gyri and hippocampus (Fig. 6; for cluster statistics, see Supplementary Table S1).

 For *love of nature*, we identified activation in partially different regions than for interpersonal love. Notably, we found activation in fusiform and parahippocampal gyri, superior and inferior parietal lobes, and cuneus, that were absent for interpersonal love. In addition, activation was found during stories in mostly medial superior, middle, and inferior frontal gyri, orbitofrontal gyri, precuneus, cingulate cortices, in subcortical regions (including amygdala and hippocampus and parts of striatum and thalamus), and cerebellum. In brainstem, activations were found in the midbrain, but these activations were smaller and more local than for the closest interpersonal relationships, which activated larger parts of midbrain. During imagery, activations were found in fusiform and parahippocampal gyri, superior parietal lobule, precuneus, cingulate cortex, cuneus, hippocampus, and cerebellum.

### Differences between types of love

**Fig. 7:**
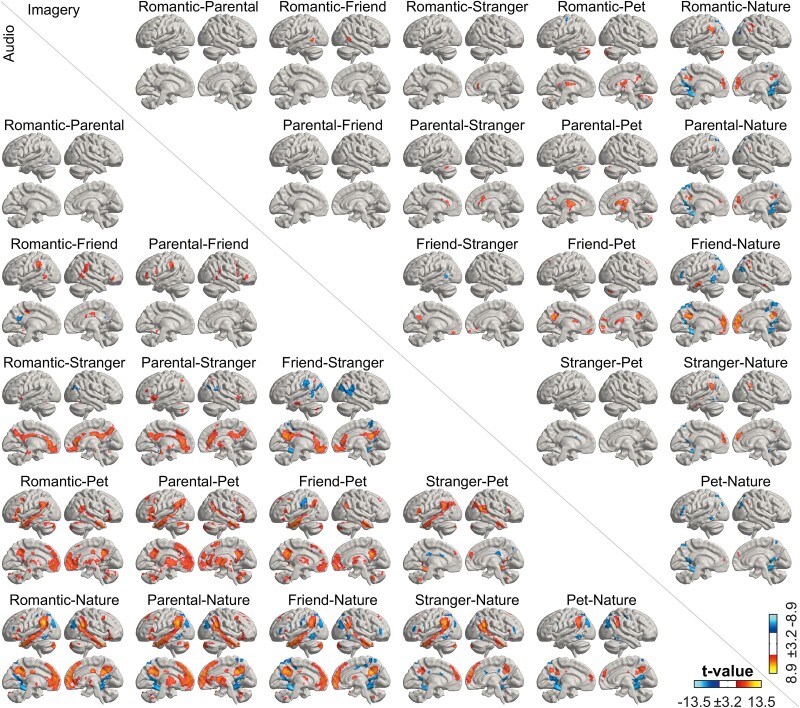
Pairwise contrasts between different types of love for audio (lower triangle) and imagery (upper triangle) conditions. Contrasts are thresholded at *P*_voxel_ < 0.001, *P*_cluster_ < 0.05 FWER. Color scales for audio and imagery conditions are indicated at the bottom right corner.

To systematically evaluate the differences between categories of love, we calculated contrasts of the six types of love with each other in a pairwise manner (Fig. 7). These contrasts highlighted clear differences between close interpersonal relationships, particularly romantic and parental love, and more distant types of love towards strangers, pets and nature. Romantic and parental love activated midline (precuneus/posterior cingulate and medial prefrontal cortex) significantly more than love for strangers, pets, and nature. Love for humans activated midline and temporoparietal regions more than love for pets and nature, particularly for the closest human relationships, while contrasts were weaker for strangers. In the cerebral cortex and subcortical regions, similar effects of closer vs. more distant types of love were observed in both audio and imagery conditions, but the differences were stronger in the audio condition. Additionally, in the auditory condition, several brainstem regions (see Supplementary Fig. S2 right column) were significantly more activated by romantic and parental love than love for strangers, pets, or nature, while love for friends vs. pets more strongly activated only a small set of brainstem regions. In the imagery conditions, only a few nuclei showed significant effects for romantic and parental love compared to love for pets while other paired contrasts showed no significant differences in the brainstem. Finally, in the opposing contrasts (distant > close), love for nature more strongly activated anterior ventral occipitotemporal and parietal regions compared to love for humans or pets, and similar effect was seen in favor of love for strangers compared to friends.

## Discussion

Utilizing a broad thematic outlook on the concept of love and naturalistic stimuli (see Jääskeläinen et al. 2021; Saarimäki 2021), we demonstrate here that feelings of love for six different object types are differentially associated with brain areas involved in processing reward and social cognition.

Previous neuroimaging studies have consistently shown that feelings of romantic and maternal love recruit subcortical brain regions associated with reward, attachment, motivation, and reinforcement learning (Bartels and Zeki 2000; Bartels and Zeki 2004; Aron et al. 2005; Fisher et al. 2005; Noriuchi et al. 2008; Acevedo et al. 2012; Diamond and Dickenson 2012; Shih et al. 2022). These areas are also implicated in pair bonding and parental attachment processes in other mammalian species such as monogamous prairie voles (Winslow et al. 1993; Curtis and Wang 2003; Tabbaa et al. 2016; Blumenthal and Young 2023). However, prior neuroimaging studies of human love have rarely expanded beyond the romantic and maternal types and have mainly relied on photographs as stimuli. A notable thematic exception is Beauregard et al. 2009, who showed with fMRI (and photographs) that “unconditional love” felt by support workers towards unknown care recipients recruits the reward system (Beauregard et al. 2009; cf. Duarte et al. 2017; Halko et al. 2017). Our results corroborate the previous findings according to which the subcortical reward system is activated during feelings of romantic and parental love. Notably, the cerebral engagement during these intimate interpersonal love types was more widespread and significantly stronger than that observed during feelings of love towards friends and strangers.

In contrast with the neutral stories, the main difference between romantic love, parental love, and love for friends in comparison with love for strangers is that the latter does not activate (or activates very little) the brainstem and shows less widespread activation in other subcortical areas associated with reward and motivation such as striatum and thalamus. This result accords with the diminished bodily and mental salience, valence, and arousal in love for strangers, in our behavioral results. Within striatum, we see activation in the nucleus accumbens, globus pallidus, and adjoining regions in the contrasts of romantic and parental love and love for friends compared to the neutral stories. In the brainstem, the most consistent areas showing significantly greater activity (in three or more pairwise contrasts) for closer relationship compared to strangers, pets, or nature were the ventral tegmental area (VTA; 5 significant contrasts), substantia nigra (5 contrasts), lateral parabrachial nucleus (LPN; 4 contrasts), pedunculotegmental nucleus (PTN; 4 contrasts), subthalamic nucleus (4 contrasts), locus coeruleus (LC; 3 contrasts), pontine reticular nucleus (3 contrasts), and red nucleus (3 contrasts). However, as the smallest of these nuclei (VTA, LPN, PTN, and LC) cover only 16 or 17 voxels at the current resolution, studies with imaging sequences optimizing the coverage, signal strength, and resolution in the brainstem would be valuable in the future to better understand the specific contributions of the brainstem nuclei to the various categories of love. We observed a similar scaling pattern from stronger to weaker interpersonal attachments in the cerebellum. That is, of interpersonal love types, love for strangers showed the least activation in the cerebellum during audio stories, and during imagery, activation in the cerebellum was found only in romantic and parental love as contrasted with control. These findings suggest that feelings of interpersonal love are modulated by activity in brain regions associated with reward according to the biobehavioral salience of the interpersonal relationship in question. Different types of interpersonal affiliation can thus be seen to form a continuum from closer affiliative bonds to more distant relationships according to the degree of subcortical and cerebellar activation.

All types of interpersonal love we investigated also recruit brain regions associated with social cognition or “theory of mind” (Gallagher and Frith 2003; Carrington and Bailey 2008; Schurz et al. 2014; Jacoby et al. 2016; Schurz et al. 2021) including midline regions in the frontal lobe, the precuneus, the middle temporal gyrus, and the intersection of the posterior superior temporal sulcus and the inferior parietal lobule (temporoparietal junction). Activations in the frontal midline regions also appear to be modulated by the closeness of the relationship, such that closer bonds activate the inferior, orbito-, middle, and superior frontal gyrus whereas the activation in love for strangers is less widespread and more superficial (see also Feldman 2017). This might suggest that ventro-frontal activations in the midline link feelings of interpersonal love to hedonic experiences, whereas temporoparietal activations may be related to processing of interpersonal relations as such (Kringelbach 2005; Parsons et al. 2013; cf. Stalnaker et al. 2015; see also Waytz et al. 2012).

It should be noted that our love for strangers condition always involved everyday altruistic behavior towards the stranger, reciprocated with an expression of gratitude. Therefore, it might be argued that the love for strangers condition is not in fact tracking love, but rather compassion or altruism. The stimuli for our love for strangers condition follow the conceptual construct of compassionate (altruistic) love by Sprecher & Fehr (2005) (see also Cassell 2021, 517). In their review of neuroscientific research on compassion, Klimecki & Singer suggest that “feelings of compassion may involve experiences of care and closeness that are similar to those invoked during feelings of love.” (Klimecki and Singer 2017, 158) They propose a common neural network for caring, feelings of social connection, and altruism. This network includes the amygdala, the ventral tegmental area, the nucleus accumbens, caudate nucleus, the pallidum, the orbitofrontal cortex, the anterior cingulate cortex, and the middle insula (Klimecki and Singer 2017, 157–158). The neural correlates of compassion provided by Stevens & Benjamin in their review of the neuroscience of compassion include the anterior insular cortex, the anterior cingulate cortex, the amygdaloid cortex, diffuse mirroring motor structures (MNS) in the PMC, IPL, PFC, posterior temporal cortex, bilateral TPJ, and precuneus. (Stevens and Benjamin 2018, 78).

It makes sense that the brain regions associated with compassion are similar to those of love in recruiting both reward and social cognition areas. There appears to be widespread consensus in contemporary interdisciplinary research on altruism that the capacity for prosocial feelings and empathy-based altruism in social groups evolved on the basis of parental nurturance (offspring care hypothesis) (see Preston 2021). As astutely pointed out by Preston, many researchers have noted an overlap between regions associated with responding to offspring and altruistic help (e.g. the nucleus accumbens, orbitofrontal cortex, thalamus, ACC), or at least a clear overlap between areas associated with altruism and reward-based decision-making. (Preston 2021, 766–767; see also Wu et al. 2021).

Our results help explain why intuitions diverge on whether compassionate love for strangers is in fact love. In harmony with the offspring care hypothesis of human altruism, our condition of love for strangers recruits similar brain regions as the more prototypical types of interpersonal love, but does so to a lesser degree in terms of reward-related activation, and is subjectively evaluated to be less salient, less pleasurable, less arousing, and *less love* according to our behavioral results (Fig. 1).

In comparison with interpersonal love types, we observed less subcortical activation in love for pets and the activation of the “theory of mind” related brain areas (frontal midline, precuneus, temporoparietal junction) did not emerge. Notably, against control we observed significant activation in the anterior cingulate gyrus. These results may be partly explained by the fact that only 27/55 of our subjects were pet owners. When we contrasted the brain activity of pet owners with non-pet owners during love for pets stories, we found activation in the precuneus/posterior cingulate cortex and left temporoparietal junction, which may indicate increased emotion processing (see Saarimäki et al. 2018) and theory of mind activity. These findings are similar to those of Hayama et al. (2016), who discovered that an owner’s subjective attachment to their pet correlates with neural activation during the viewing of owned pet photographs in the precuneus, cuneus, and superior parietal lobule. Our result suggests that for pet owners, love for pets is neurally more similar to interpersonal love than for participants without pets.

In line with our general prediction of differential recruitment of social cognition areas for different types of love, love for beautiful nature also did not activate all regions typically associated with social cognition. Notably, and in contrast with interpersonal love types, TPJ activations were missing. Instead, we found activation in the parahippocampal gyrus, which has previously been associated with the viewing of landscapes in particular (Epstein and Kanwisher 1998; Epstein et al. 2009; Häusler et al. 2022). Interestingly, and like in other types of love (excluding love for strangers), we further found significant activation in the anterior cingulate gyrus against control. This region has been previously associated with multiple functions (Chudasama et al. 2013; Tang et al. 2015; Monosov 2017; Stevens and Benjamin 2018; Lockwood et al. 2020; Preston 2021) and is included in generic neurobiological frameworks of human affiliation for its association with reward, salience, and social cognition in close relationships (Feldman 2017; Bortolini et al. 2024).

The activations we found during the imagery period were mainly similar but weaker than in the audio condition. In particular, the temporal and parietal lobes deactivated across types of love. The remaining activations can be thought to represent the mere imagined feelings of love for the objects (discounting the naturalistic phenomenological contexts of the audio narratives).

While our cohort of participants is to date the largest in a neuroscientific study on love, the generalizability of our results is limited by the demographics of our sample. Love is a complex and multifaceted set of biologically grounded and culturally modified phenomena, and further cross-cultural research is still required for a better understanding of how cultural and demographic factors influence various feelings of love and their correlates in the human brain.

In conclusion, our results show how the object of love modulates the feeling of love and its neural basis. The activation of brain areas associated with social cognition is common to varieties of interpersonal love. Closer affiliative bonds elicit stronger feelings of love, which are associated with more activation in the reward pathways. Our research offers insights into why we feel stronger affection for those we are close to compared to strangers, even though the underlying brain processes of affection are the same for all types of interpersonal relationships. This may help explain why religions and philosophical traditions such as Christianity or Buddhism refer to benevolence towards others as “neighborly love” or “loving-kindness,” even if it does not feel as intense as the love we have for close connections. The graded recruitment of similar reward regions across types of interpersonal love coheres with the offspring care hypothesis of human altruism. A subjectively closer affiliation with a nonhuman animal (pet owner) makes interspecies love neurally more resemblant of interpersonal love. Together with the fact that subjective salience influences feelings of love and their neural substrates, the involvement of reward regions in interspecies love and love for nature (ACC) supports the view that different types of love fall on a “fuzzy” continuum (Fehr and Russell 1991; Rinne et al. 2023; see also Cowen and Keltner 2017) where romantic love (pair bonding) and parental love (parental care) are the prototype cases, and compassionate love for unknown conspecifics, interspecies love, and more abstract types of nonsocial love resemble the prototypes in varying degrees dependent on biological, cultural, and subjective psychological influences. From the perspective of functional neuroarchitecture, the wonderful complexity of human love can be seen as emerging from and building on fundamentally adaptive biological attachment systems shared with other mammals.

## Supplementary Material

Supplement_Revised_9July2024_bhae331

Supplementary_Tables_bhae331
