# Supplementary material for: Six types of loves differentially recruit reward and social cognition brain areas
Source: Cereb Cortex. 2024 Aug 26;34(8):bhae331. doi: 10.1093/cercor/bhae331 (PMC11345515; doi:10.1093/cercor/bhae331)
Supplement: Supplement_Revised_9July2024_bhae331 [file supplement_revised_9july2024_bhae331.pdf]

Six types of loves differentially recruit reward and social cognition brain areas

Supplementary Information

Pärttyli Rinne, Juha Lahnakoski, Heini Saarimäki, Mikke Tavast, Mikko Sams, Linda Henriksson

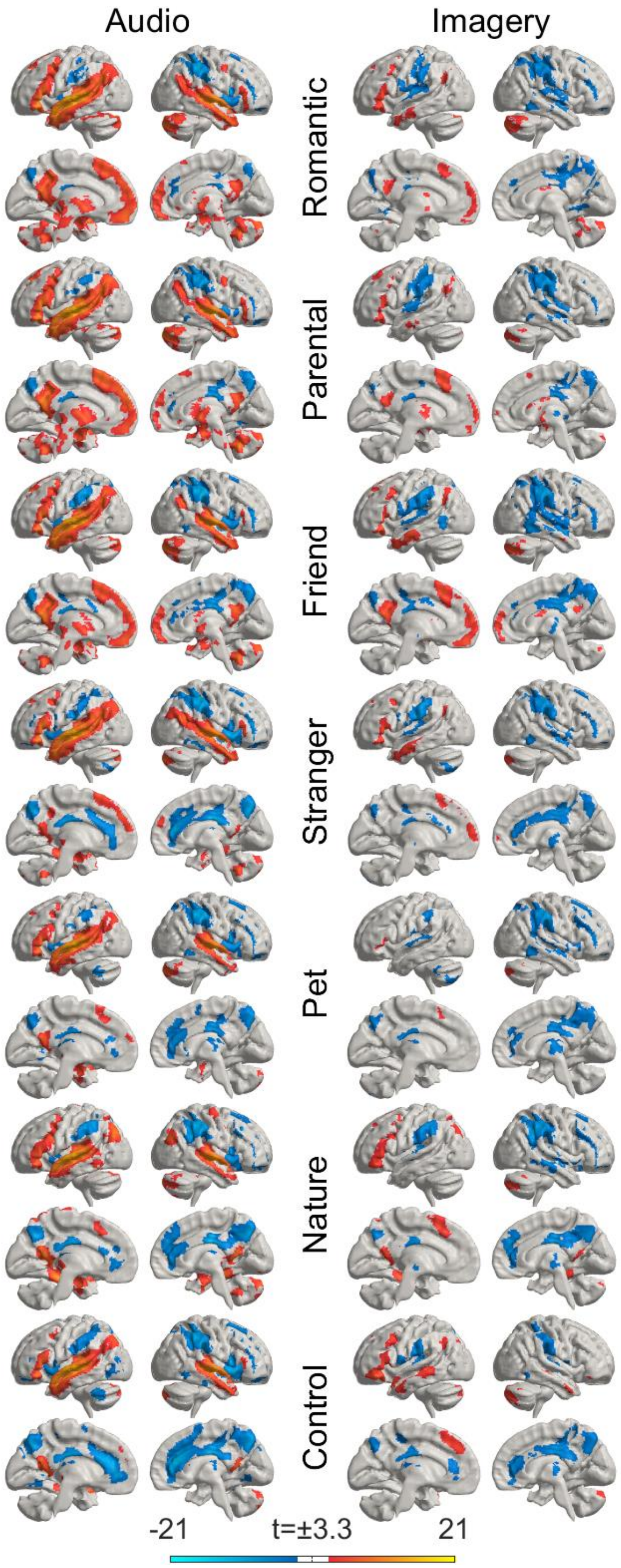

Supplementary Figure S1. Main effects for individual categories of love and the control stories during audio (left) and imagery (right) conditions. Hot color indicate activations compared to baseline, while cold colors indicate deactivations.

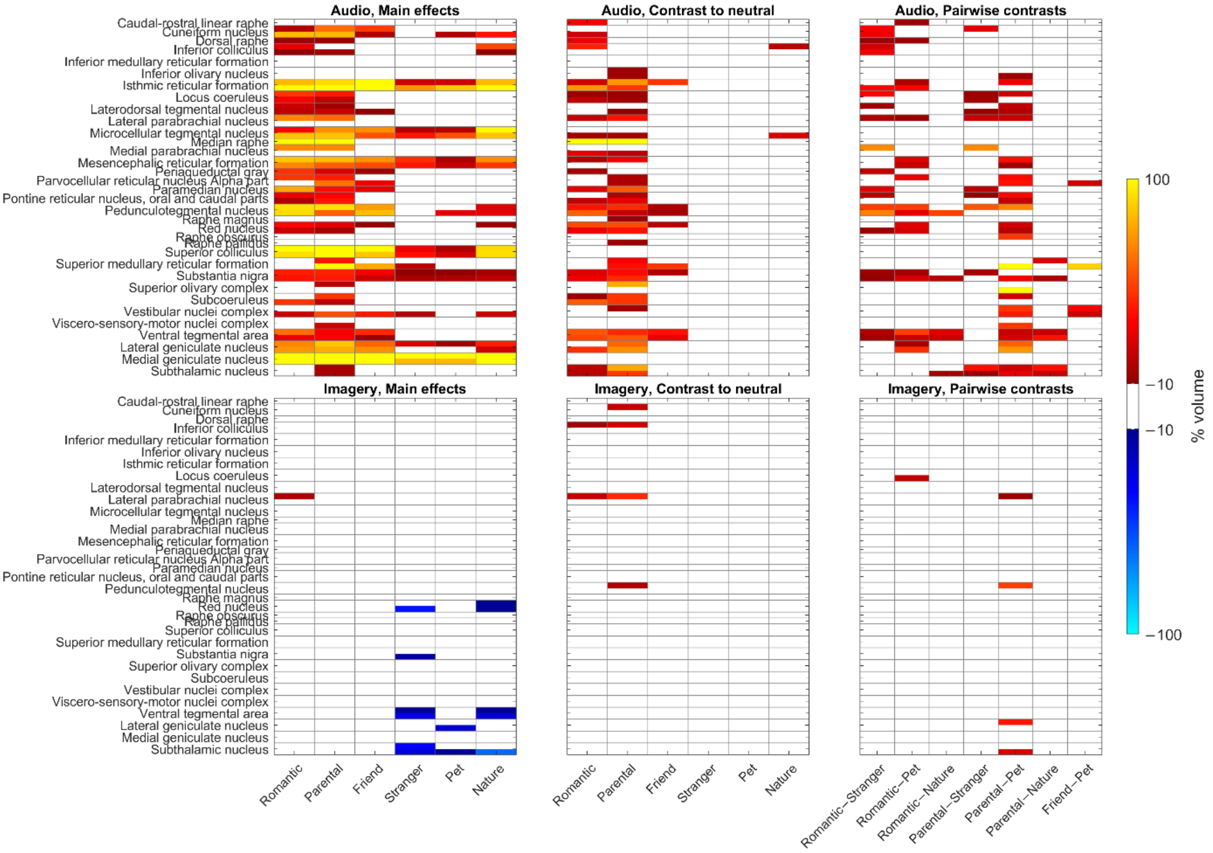

Supplementary Figure S2. Regions of the brainstem and diencephalon activated by listening (top) and imagery (bottom). Left column shows the main effects compared to baseline, middle column shows contrasts to neutral stories, and right column shows pairwise contrasts between types of love. Pairwise contrasts with no significant effects are excluded. Nuclei with at least 10% of voxels activated are colour coded based on the total percentage volume (by voxel count) showing significant (de)activation.

Supplementary Table S1*. Areas showing higher activity for love for pets in pet owners than non-owners. The t-values correspond to local cluster maxima, region labels correspond to the Brainnetome names and coordinates are listed in millimeters in the MNI space.*

Cluster size	t-value	Region	X	Y	Z
134 voxels	3.60	PhG_L_6_3	-30	-28	-22
	4.41	Hipp_L_2_2	-30	-34	-12
	4.28	PhG_L_6_6	-22	-40	-10
	4.25	PhG_L_6_3	-28	-38	-10
	4.18	Hipp_L_2_2	-26	-34	-6
126 voxels	3.59	MTG_L_4_3	-52	-52	2
	4.42	pSTS_L_2_2	-48	-48	8
	3.92	pSTS_L_2_2	-60	-50	12
671 voxels	4.39	Pcun_L_4_3	-16	-56	16
	4.63	Pcun_L_4_4	-8	-54	16
	4.23	CG_R_7_4	6	-48	16
	4.53	Pcun_R_4_3	10	-54	18
	3.59	CG_L_7_4	-8	-46	18
	4.49	Pcun_R_4_4	6	-58	24
	4.25	Pcun_L_4_3	-16	-66	26
	4.19	Pcun_R_4_4	4	-62	28
	3.93		18	-54	28
	3.65	Pcun_L_4_3	-8	-64	30
	3.78	Pcun_L_4_4	-4	-64	30
	4.22	Pcun_R_4_4	10	-54	30
	3.69	Pcun_L_4_4	-2	-62	34
171 voxels	4.98	IPL_L_6_1	-32	-78	42
	3.97	sOcG_L_2_2	-20	-78	48

Online experiment

Procedure

In the online experiment, the participants rated random subsets of textual versions of the 42 stimuli used in the fMRI experiment. The ratings were conducted in sets of 14 stimuli. After each set, participants were asked if they wanted to continue the rating task or exit the experiment. Thus, each participant could have rated one, two, or three sets of stimuli, however, we only included in the final analyzes participants who completed at least one set of 14 ratings.

In the experiment, the set of 14 stimuli were presented one-by-one, in a random order. The participants were asked to read to text and immerse themselves in the situation and the feelings that the text elicits. Their task was to rate their experiences in the same 7 visual analogue scales as described in the behavioral task of the fMRI experiment: 1) “How strongly does the feeling elicited by the stories feel in the body” (*not at all – very much*) 2) “How strongly does the feeling elicited by the stories feel in the mind” (*not at all – very much*) 3) “How pleasant was

the feeling elicited by the stories" (*extremely unpleasant – extremely pleasant*) 4) "How arousing was the feeling elicited by the stories" (*calm – excited*) 5) "How often do you feel a feeling that is similar to the one elicited by the stories?" (*never - often*) 6) "How well do the feelings elicited by the stories correspond with your own conception of what love is?" (*not at all – extremely well*) 7) "How easy was it for you to immerse yourself in the situations described in the stories?" (*extremely hard – extremely easy*). The scale values were arbitrarily set to range from 0 to 1000.

Participants

In total, 281 participants registered for the experiment, and 189 completed at least one set of 14 ratings. We excluded 8 participants as non-native Finnish speakers from the final analyzes. Thus, the final sample size was 181. On average, each stimulus was rated by 82.5 participants (range 68-101). The study was advertised in student email lists. After the experiment, the participants could attend a raffle, where they could win a 30€ Aalto University Shop Gift Card.

Results

The means and standard errors for each stimulus are presented in Supplementary Figure S3.

Discussion

Similar to the fMRI experiment, *romantic love* stimuli were rated to be strongly experienced in the mind and in the body. However, in contrast with the fMRI experiment, *parental love* stimuli were reported to be amongst the most difficult to imagine and least frequently experienced. This is most likely explained by the fact that in the online sample, only 11.0% of participants were parents (in the fMRI experiment all participants were parents). Prototypical love types of *romantic love* and *parental love* corresponded strongly to the participants own understanding of love. *Love of nature* stimuli were reported to be relatively high in valence, but low in arousal.

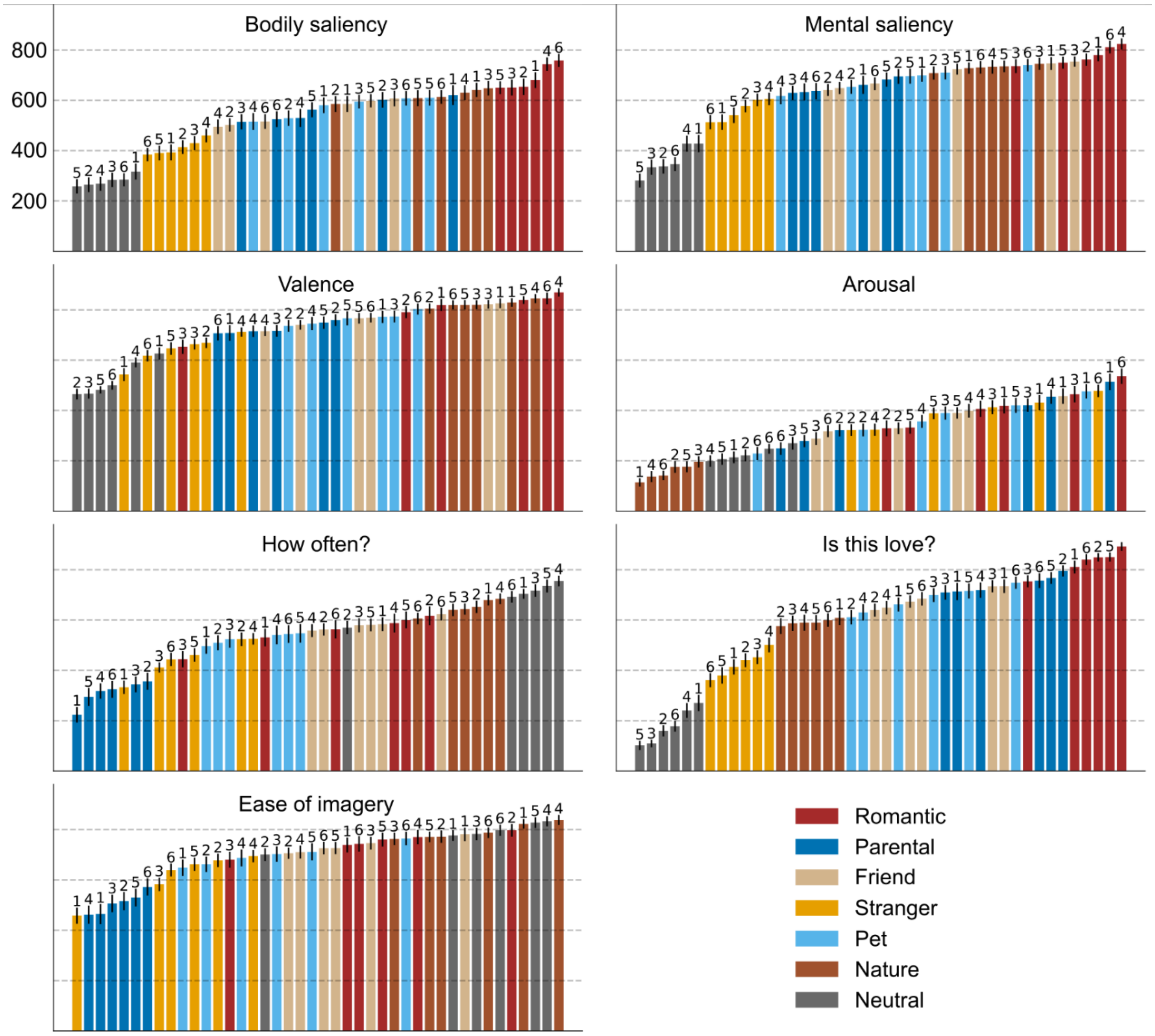

Supplementary Figure S3. Means and standard errors of the mean for each stimulus from the online experiment. The numbers above the bars indicate the stimulus number, the stimuli can be found at the end of this supplementary file.

Supplementary Table S2. Participant demographics of the online experiment.

Question	Items	Frequency	Percentage
Gender	Man	33	18.2%
	Woman	138	76.2%
	Other or do not want to define	10	5.5%
Age	18-25	101	55.8%
	26-35	53	29.3%
	36-45	15	8.3%
	46-55	9	5.0%
	56-65	2	1.1%
	66 or more	1	0.6%
Do you have children?	No	161	89.0%
	Yes	20	11.0%
	Do not want to answer	0	0%
Are you in a relationship?	No	64	35.4%
	Yes	113	62.4%
	Do not want to answer	4	2.2%
Do you have pets?	No	115	63.5%
	Yes	66	36.5%
	Do not want to answer	0	0%

Stimuli: Finnish language narratives and their English translations in parallel text format

Note: The Finnish language does not have gendered pronouns, and the third person singular pronoun ‘hän’ (frequently used in the stories to refer to an object of love) can mean she/he/they, depending on the context. As the stories are designed to evoke the personal experiences of the experiment subjects with reference to any sex/gender they may have in mind when thinking about an object of interpersonal love, in the English translations ‘hän’ is consistently rendered ‘they’ (without implying any *added* emphasis on gender neutrality in the original stories).

1. Romanttinen rakkaus / Romantic love

1.1. Olet kynttiläillallisella kumppanisi kanssa. Katsot häntä silmiin pöydän yli, ja välillänne vallitsee sanaton yhteis-ymmärrys. Rakastat kumppaniasi.

1.1. You are having a candle lit dinner with your partner. You look into their eyes over the table, and you share a mutual understanding without words. You love your partner.

1.2. Katselet kumppaniasi arkitoimissaan. Hän pukee hajamielisenä paitaa ylleen. Tunnet rakkautta häntä kohtaan.

1.2. You are watching your partner in their daily routines. They are absentmindedly putting on a shirt. You feel love for them.

1.3. Olet riidellyt kumppanisi kanssa, mutta riita on sovittu ja olette pyytäneet toisiltanne anteeksi. Katselet hänen kasvojaan, ja tunnet rakkautta häntä kohtaan.

1.3. You have had an argument with your partner, but you have made up and apologised to each other. You look at their face, and you feel love for them.

1.4. Olet ollut kumppanisi kanssa yhdessä jo monta vuotta. Näet yhä hänen kasvoillaan saman ilon ja leikin kuin nuorempana, ja haluat olla hänen kanssaan kuolemaasi asti. Rakastat häntä.

1.4. You have been together with your partner for many years already. You still see the same joy and playfulness on their face as when you were younger, and you want to be with them until you die. You love them.

1.5. Olet kumppanisi kanssa pyykkituvassa. Hän nostelee pyykkejä koneeseen, ja yhtäkkiä muistat taas kuinka ihana ihminen kumppanisi onkaan. Tunnet rakkautta häntä kohtaan.

1.5. You are in the laundry room with your partner. They are loading the washer with laundry, and suddenly you remember what a lovely person your partner is. You feel love for them.

1.6. Olet kävelyllä kumppanisi kanssa. Hän pysähtyy ja kääntyy katsomaan sinua syvälle silmiin pehmeästi hymyillen. Rakastat häntä.

1.6. You are taking a walk with your partner. They stop and turn to look at you deep in the eyes with a soft smile. You love them.

2. Vanhempain rakkaus / Parental love

2.1. Näet vastasyntyneen lapsesi ensimmäistä kertaa. Vauva on pehmeä, terve ja elinvoimainen - elämäsi suurin ihme. Tunnet rakkautta pienokaista kohtaan.

2.1. You see your newborn child for the first time. The baby is soft, healthy and hearty – your life’s greatest wonder. You feel love for the little one.

2.2. Lapsesi juoksee iloisena luoksesi aurinkoisella niityllä. Hymyilette yhdessä ja auringon säteet välkkyvät hänen kasvoillaan. Tunnet rakkautta lastasi kohtaan.

2.2. Your child runs to you joyful on a sunny meadow. You smile together and the sunrays flicker on their face. You feel love for your child.

2.3. Olet lapsesi kanssa huvipuistossa. Lapsi rynnii riemuiten laitteesta toiseen, ja olet iloinen kun saat todistaa hänen onneaan. Rakastat lastasi.

2.3. You are in an amusement park with your child. The child is rushing with glee from one ride to the next, and you are joyful for getting to witness their happiness. You love your child.

2.4. Lapsesi valmistuu ylioppilaaksi. Olet ylpeä hänestä, ja tiedät että mihin ikinä maailma hänet viekin, tulet rakastamaan lastasi.

2.4. Your child graduates from high school. You are proud of them, and you know that where ever the world may lead them, you will love your child.

2.5. Lapsesi istuu kotona tietokoneen ääressä. Hän kääntää katseensa sinuun ja hymyilee. Tuntuu hyvältä saada elää hänen kanssaan – rakastat lastasi.

2.5. Your child is home sitting at the computer. They turn their eyes to you and smile. It feels good to get to live with them – you love your child.

2.6. Olet metsäretkellä lapsesi kanssa ja pysähdytte syömään eväitä. Lapsesi juo tyytyväisenä pillimehua ja haukkaa voileipää. Tunnet rakkautta häntä kohtaan.

2.6. You are on a forest trip with your child and you stop for snacks. Your child is sipping on a juice box and biting a sandwich. You feel love for them.

3. Rakkaus ystäviin / Love for friends

3.1. Paras ystäväsi on palannut pitkältä matkalta ja tapaat hänet kahvilassa. Ette ole nähneet yli vuoteen, mutta jo heti tervehtiessä tunnet, että välillänne on sama yhteys kuin ennenkin. Rakastat ystävääsi.

3.1. Your best friend has returned from a long trip and you meet them at a cafe. You have not seen each other for over a year, but immediately as you greet, you feel that there is the same connection between you as before. You love your friend.

3.2. Tarvitset apua muutossa ja soitat ystävällesi. Hän lupaa tietenkin tulla auttamaan, ja pian nostelette muuttolaatikoita yhdessä pakettiautoon. Arkisen tilanteen keskellä tunnet rakkautta ystävääsi kohtaan.

3.2. You need help moving house and you call your friend. They promise to of course come to help out, and soon you are lifting cardboard boxes together in a van. In the middle of the ordinary situation you feel love for your friend.

3.3. Ystäväsi on luonasi kylässä ja valvotte yhdessä aamuun asti keskustellen. Tiedät, että voit aina luottaa häneen. Rakastat ystävääsi.

3.3. Your friend is visiting you at your place, and you stay up together conversing until morning. You know you can always trust them. You love your friend.

3.4. Ystäväsi tarvitsee apua tavaroiden kuljettamisessa kesämökilleen. Raivaat kalenteriin tilaa, ja ajatus yhteisestä ajomatkasta saa sinut hymyilemään. Tunnet rakkautta ystävääsi kohtaan.

3.4. Your friend needs help transporting things to their summer cottage. You make room in your calendar, and the thought of the joint car ride makes you smile. You feel love for your friend.

3.5. Laitatte yhdessä ruokaa ystäväsi kanssa. Radiossa soi popmusiikki ja ystäväsi laulaa hullunkurisesti kappaleen mukana. Sinun on hyvä olla hänen lähellään – rakastat ystävääsi.

3.5. You are cooking together with your friend. The radio is playing pop music, and your friend is singing funnily along with the tune. You feel good being near them – you love your friend.

3.6. Olet ystäväsi kanssa taidemuseossa. Ystäväsi kommentoi teoksia rennosti vitsaillen ja hymyilee samalla sinulle. Tunnet rakkautta ystävääsi kohtaan.

3.6. You are at an art museum with your friend. Your friend comments on the works making casual jokes while smiling at you. You feel love for your friend.

4. Rakkaus muukalaisiin / Love for strangers

4.1. Näet lumisella kadulla vanhan kerjäläisnaisen. Haluat auttaa ja annat naiselle taskusi pohjalta löytyvät lantit. Hän kiittää sinua hymyillen, ja katseidenne kohdatessa tunnet rakkautta kerjäläisnaista kohtaan.

4.1. You see an old beggar woman on a snowy street. You want to help and you give the woman the coins from the bottom of your pocket. She thanks you smiling, and when your eyes meet you feel love for the beggar woman.

4.2. Näet perheenäidin kamppailevan ruokakaupan oven välissä lastenvaunujen kanssa. Pitelet ovea auki että hän pääsee vaunuineen sisälle. Perheenäiti hymyilee sinulle, ja tunnet rakkautta häntä kohtaan.

4.2. You see a mother caught in the door of a grocery store, struggling with a baby carriage. You hold the door open so she can enter with the carriage. The mother smiles at you, and you feel love for her.

4.3. Kehitysvammainen mies pyrkii vilkasliikenteisen tien yli. Viittilöit autoja pysähtymään ja autat miehen tien toiselle puolen. Hän näyttää iloiselta, ja tunnet rakkautta häntä kohtaan.

4.3. A developmentally disabled man is trying to cross a busy road. You wave for the cars to stop and help the man across the street. He looks joyful, and you feel love for him.

4.4. Näet kadulla vanhan naisen raahaavan raskaita kauppakasseja. Autat häntä kantamalla yhden kasseista korttelin päähän hänen kotiovelleen. Vanha nainen on kiitollinen, ja tunnet rakkautta häntä kohtaan.

4.4. You see an old woman on the street carrying heavy grocery bags. You help her by carrying one of the bags to her home door one block away. The old woman is grateful, and you feel love for her.

4.5. Aasialaistaustainen miesturisti tutkii kadunkulmassa eksyneen näköisenä kännykän karttaa. Neuvot miehelle tien hänen etsimäänsä museoon ja hän kiittelee sinua vuolaasti. Tunnet rakkautta häntä kohtaan.

4.5. A male tourist of Asian background is at a street corner investigating the map of his mobile phone, looking lost. You tell the man the way to the museum he is looking for and he thanks you at length. You feel love for him.

4.6. Istut lähijunassa ja näet junasta poistuvan miehen unohtavan hansikkaansa penkille. Kiiruhdat ojentamaan miehelle hänen hanskansa. Hän kiittää sinua hymyillen, ja tunnet rakkautta tuntematonta miestä kohtaan.

4.6. You are sitting on a local train and you see a man exiting the train forget his gloves on the seat. You rush to hand the man his gloves. He thanks you with a smile, and you feel love for the unknown man.

5. Rakkaus lemmikkieläimiin / Love for pets

5.1. Perheessänne on uusi koiranpentu. Pikkuinen koira heiluttaa häntäänsä, nuuskii innokkaasti ja tassuttelee vilkkaana sinne tänne kotinne lattioilla. Tunnet rakkautta suloista koiranpentua kohtaan.

5.1. There is a new puppy in your family. The little dog wags its tail, sniffs around eagerly and pads vivaciously hither and tither on the floors of your home. You feel love for the cute puppy.

5.2. Perheessänne on uusi kissanpentu. Pikkuinen kissa latkii maitoa, kehrää ja katsoo sinua suurilla silmillään. Tunnet rakkautta suloista kissanpentua kohtaan.

5.2. There is a new kitten in your family. The little cat laps milk, purrs and looks at you with its wide eyes. You feel love for the cute kitten.

5.3. Olet puistossa leikkimässä koirasi kanssa. Heität koiralle keppiä ja tämä noutaa sen innokkaasti häntäänsä heiluttaen. Rakastat koiraasi.

5.3. You are in a park playing with your dog. You toss a stick for the dog and it retrieves it enthusiastically wagging its tail. You love your dog.

5.4. Leikit kissasi kanssa kotonanne. Pyörität lankakerää lattialla ja leikkisä kissa vaanii ja hyökkäilee lankakerän kimppuun. Rakastat kissaasi.

5.4. You are home playing with your cat. You roll a ball of yarn on the floor and the playful cat prowls and attacks the yarn. You love your cat.

5.5. Tulet kotiin töistä ja lemmikkikoirasi rientää ovelle tervehtimään sinua. Se heiluttaa häntäänsä, nuuskii ja läähättää innostuneena nähdessään sinut. Rakastat lemmikkiäsi.

5.5. You come home from work and your pet dog hurries to the door to greet you. It wags its tail, sniffs and pants excitedly upon seeing you. You love your pet.

5.6. Loikoilet kotonasi sohvalla ja lemmikkikissasi tassuttelee luoksesi. Kissa käpertyy viereesi kerälle ja kehrää uneliaana. Rakastat lemmikkiäsi.

5.6. You are home lolling on the couch and your pet cat pads to you. The cat curls up next to you and purrs sleepily. You love your pet.

6. Rakkaus luontoon / Love for nature

6.1. Olet mökilläsi metsän keskellä. Ympärilläsi on puiden vihreys, ilma on raikas ja tuuli suhisee kevyesti koivujen lehvissä. Tunnet rakkautta luontoa kohtaan.

6.1. You are at your cottage in the middle of the woods. Around you is the greenness of trees, the air is fresh and the wind blows gently through the birch leaves. You feel love for nature.

6.2. Olet kävelyllä luonnonpuistossa. Linnut laulavat kuusten oksilla, puro solisee alas jyrkkiä kallioita ja edessäsi aukeaa suomalainen järvimaisema. Tunnet rakkautta luontoa kohtaan.

6.2. You are taking a walk in a natural park. The birds are singing on the branches of spruce trees, a brook burbles down steep cliffs and a Finnish lake landscape opens up in front of you. You feel love for nature.

6.3. Seisot lumisen tunturin laella. Aurinko värjää taivaan kultaan ja purppuraan, kaukana horisontissa on metsää, ja alapuolellasi puut nuokkuvat tykkylumesta valkeina. Rakastat ympäröivää luontoa.

6.3. You are standing on top of a snowy fjeld. The sun paints the sky in gold and purple, there is forest far away on the horizon, and below you trees are weeping white of crown snow load. You love the surrounding nature.

6.4. Olet saaristossa meren rannalla. Siniset aallot liplattavat rantakiviin, vieressäsi kohoaa käppyrämänty, ja taivaalla on siellä täällä valkeita pilven hattaroita. Rakastat luontoa.

6.4. You are in the archipelago at the seaside. The blue waves ripple over the coastal stones, a crooked pine rises next to you, and there are white fluffy clouds here and there in the sky. You love nature.

6.5. Seisot korkealla kukkulalla. Aurinko paistaa, horisontissa aukeaa avoin, sininen taivas, ja alhaalla laaksossa on vehreä niitty, jonka keskeltä virtaa raikkaana soliseva puro. Rakastat luontoa.

6.5. You are standing on a high hill. The sun is shining, the clear, blue sky opens up on the horizon, and there is a verdant meadow down in the valley, through which a fresh, burbling brook flows. You love nature.

6.6. Soutelet tyynellä, sateisella järvellä. Airot halkovat veden pintaa, ilmassa on pehmeää usvaa ja kevyttä tihkua, jossain kaukana kuikkalinnun huuto. Tunnet rakkautta luontoa kohtaan.

6.6. You are rowing on a placid, rainy lake. The oars are cutting through the surface of the water, there is soft mist and light drizzle in the air, the scream of a diver bird somewhere far away. You feel love for nature.

7. Neutraali / Neutral

7.1. Kävelet kadulla kotisi lähellä. Liikenne on vähäistä ja siellä täällä kulkee jokunen ihminen. Mielessäsi ei pyöri mitään sen kummempaa.

7.1. You are walking on the street near your home. There is scarce traffic and a few people going about here and there. There is nothing in particular on your mind.

7.2. Istut autossa matkalla töihin. Radion säätiedotuksessa sanotaan, että lauhaan säähän ei ole lähipäivinä tulossa muutoksia. Työpaikallakaan ei tapahdu juuri nyt oikein mitään.

7.2. You are sitting in the car going to work. The weather forecast on the radio says, that the mild weather is not about to change for the next few days. There's not much going on at the work place either.

7.3. Istut kotona sohvalla ja syöt hajamielisenä voileipää. Leipä on tavanomainen, eikä sen maku ole hyvä eikä paha. Ajatuksesi harhailevat.

7.3. You are sitting on the sofa at home and absentmindedly eating a sandwich. The sandwich is ordinary, and it tastes neither good nor bad. Your mind is wandering.

7.4. Olet bussissa matkalla kotiin. Bussin ikkunasta näkyy taloja, autoja ja kadulla käveleviä ihmisiä. Näkymä on sangen tavallinen.

7.4. You are on the bus going home. Through the window of the bus one can see houses, cars and people walking on the street. The view is quite ordinary.

7.5. Harjaat hajamielisenä hampaita. Harja liikkuu suussasi vähän kuin automaattisesti. Et kiinnitä toimenpiteeseen erityisempää huomiota.

7.5. You are absentmindedly brushing your teeth. The brush is moving in your mouth as if automatically. You are not paying any special attention to the procedure.

7.6 Solmit kengännauhoja kotisi eteisessä. Olet lähdössä ruokakauppaan. Kauppaan meno tuntuu arkiselta ja jokapäiväiseltä, eikä mielessäsi ole mitään erityisiä ajatuksia tai voimakkaita tuntemuksia.

7.6. You are tying your shoelaces in the hallway of your home. You are leaving for the grocery store. Going to the store feels mundane and common, and there are no specific thoughts or strong sensations in your mind.
